# Nonlinear association between blood cadmium levels and periodontitis: a cross-sectional study from NHANES 2011–2014

**DOI:** 10.1038/s41405-025-00376-y

**Published:** 2025-11-13

**Authors:** Chong Gao, Ning Sun, Ludan Xu, Zhengchuan Zhu, Qiuyan Li, Miaoran Wang

**Affiliations:** 1 Department of Pediatric dentistry, School and Hospital of Stomatology, Wenzhou Medical University, Wenzhou, China; 2 Xiyuan Hospital, China Academy of Chinese Medical Sciences, Beijing, China

**Keywords:** Periodontitis, Dental epidemiology

## Abstract

**Objectives:**

The relationship between blood cadmium levels and the risk of periodontitis remains unclear. This study aimed to explore the association between blood cadmium concentrations and periodontitis in a large sample of the U.S. population from 2011 to 2014.

**Methods:**

This cross-sectional study analyzed data from 5215 participants in the National Health and Nutrition Examination Survey (NHANES) 2011–2014. Blood cadmium levels were the exposure variable, and periodontitis was the outcome variable. Multivariate logistic regression and a two-piecewise linear regression model were used to examine the nonlinear relationship between blood cadmium levels and periodontitis. Stratified analyses were conducted to identify subgroups at higher risk.

**Results:**

The study identified a nonlinear relationship between blood cadmium levels and periodontitis risk. The threshold effect was observed at 0.37 µg/L and 1.20 µg/L. When blood cadmium levels were below 0.37 µg/L, the odds ratio (OR) for periodontitis was 0.88 (95% CI: 0.31, 2.48; p = 0.81). For cadmium levels between 0.37 and 1.20 µg/L, the OR increased significantly to 12.40 (95% CI: 2.77, 55.57; p < 0.001). When cadmium levels exceeded 1.20 µg/L, the OR decreased to 0.45 (95% CI: 0.06, 3.39; p = 0.44).

**Conclusions:**

The study found a nonlinear association between blood cadmium levels and periodontitis risk in U.S. adults. The risk of periodontitis increased significantly when blood cadmium levels were between 0.37 and 1.20 µg/L.

## Introduction

Periodontitis is a chronic inflammatory disease that primarily affects the gums and bone supporting the teeth, leading to significant tissue destruction and, if untreated, tooth loss [1]. This condition poses a major public health challenge, with approximately 46% of U.S. adults affected, and severe periodontitis impacting about 8.9% of the population [2]. The disease is not limited to oral health; it has been linked to various systemic diseases, including diabetes, cardiovascular diseases, and neurodegenerative disorders such as Alzheimer’s disease [3, 4].

Cadmium is a highly toxic heavy metal that poses significant health risks due to its widespread presence in the environment, primarily as a result of industrial and agricultural activities [5–7]. It is known to accumulate in the human body, particularly in the kidneys and bones, where it can persist for decades, with a biological half-life estimated at 10–30 years [8]. This prolonged retention contributes to various adverse health effects, including osteoporosis, kidney dysfunction, and oxidative stress [9]. Recent studies suggest that cadmium may also play a role in the development of periodontitis by promoting bone resorption and inflammation [10, 11].

Despite the growing evidence of cadmium’s toxicity, the relationship between blood cadmium levels and periodontitis remains poorly understood. This study aims to fill this gap by examining the association between blood cadmium levels and periodontitis using data from the NHANES 2011–2014.

## Methods

### Study design and population

**Fig. 1:**
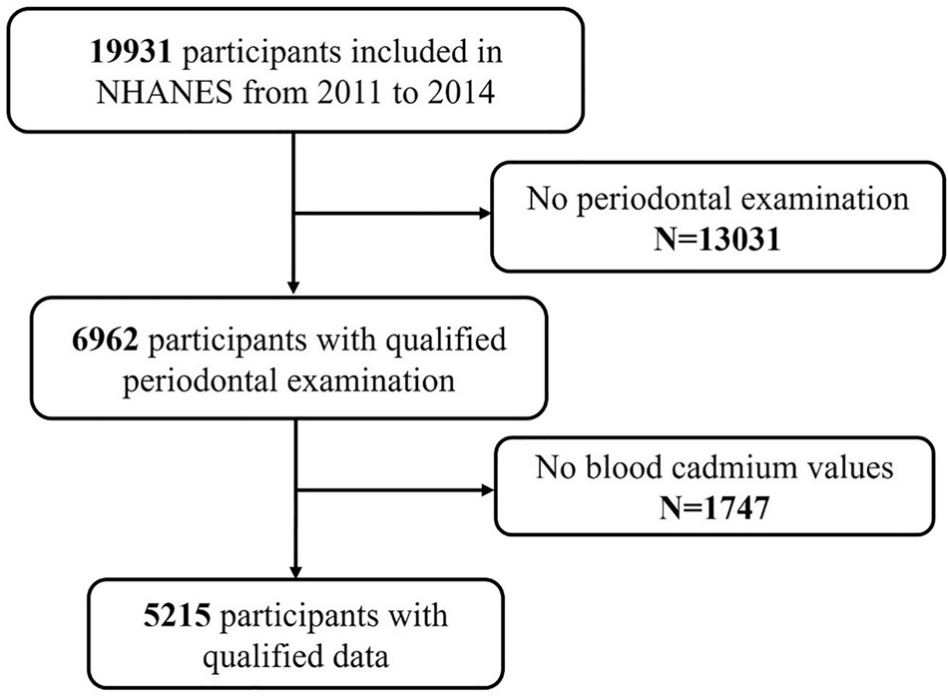
Flow chart of study participants.

The NHANES follows the STROBE guidelines [12]. This cross-sectional study utilized data from the NHANES 2011–2014, a nationally representative survey conducted by the Centers for Disease Control and Prevention. The NHANES collects demographic, dietary, and health-related data through interviews, physical examinations, and laboratory tests. Overall, 19,931 participants took part in the interview (Fig. 1). A total of 6962 people from the NHANES had complete periodontal examination records from 2011 to 2014.After excluding 1747 individuals due to missing blood cadmium information, the cross-sectional study included 5215 participants.

### Periodontitis assessment

The following steps were taken to inspect periodontal disease: According to the NHANES examination protocol, trained dental examiners conducted a full-mouth periodontal examination on participants who had at least one natural tooth [13]. Periodontitis was assessed using the CDC/AAP case definition, which classifies periodontitis into mild, moderate, and severe based on clinical attachment loss (CAL) and probing depth (PD) [14]. Participants were categorized as having periodontitis if they met the criteria for mild, moderate, or severe periodontitis [15]. Mild periodontitis was defined as having either two or more interproximal sites with CAL of at least 3 mm and PD of at least 4 mm (not on the same tooth), or one site with a PD of 5 mm or greater [16]. Moderate periodontitis is characterized by having at least two interproximal sites with CAL of 4 mm or more, or at least two interproximal sites with PD of 5 mm or more, provided these sites are not on the same tooth [15]. Severe periodontitis is defined as having at least two interproximal sites with CAL of 6 mm or more, not on the same tooth, and at least one interproximal site with a PD of 5 mm or more [15]. Individuals not classified within these categories were deemed free of periodontal disease. In our study, periodontitis was classified into two categories: yes (encompassing mild, moderate, and severe cases) and no.

### Blood cadmium measurement

Blood cadmium levels were measured using inductively coupled plasma mass spectrometry [17, 18]. The detection limit for cadmium was 0.10 µg/L. Blood cadmium levels were categorized into low (<0.10 µg/L), medium (0.10–0.24 µg/L), and high (>0.24 µg/L) according to medians.

### Covariates

According to a previous study [19–21], covariates included age, sex, race/ethnicity, education level, smoking status, poverty-income ratio (PIR), body mass index (BMI), vitamin D and history of diabetes and hypertension. Smoking status was categorized as never (fewer than 100 cigarettes in their lifetime), former (over 100 cigarettes in their lifetime but have quit), or current smoker (over 100 cigarettes in their lifetime and still smoking). The determination of previous disease (diabetes, hypertension) was based on whether the questionnaire indicated that a doctor had previously notified the patient of these issues.

### Statistical analysis

Analyses utilized sampling weights to account for the NHANES survey’s complex design. Continuous variables were expressed as mean ± standard deviation (normal distribution), or median (quartile) (skewed distribution). Categorical variables are presented as percentages (n%). The t test (normal) or one-way ANOVA, Mann-Whitney (skewed distribution) or Kruskal-Wallis H test and chi-square tests (categorical variables) were used to determine any statistical difference between the means and proportions of the cadmium groups.

A multivariate logistic regression model, utilizing generalized additive models and smoothed curve fitting, was developed to examine the association between blood cadmium levels and periodontitis risk. A two-piecewise linear regression model was employed to identify threshold effects [22]. Model 1 was unadjusted. Model 2 was adjusted for age, sex, and ethnicity. Model 3 was adjusted for variables including age, sex, ethnicity, BMI, PIR, current smoking status, vitamin D levels, diabetes, and hypertension.

To examine the robustness of the results, we conducted sensitivity analyses. Multiple imputation techniques were employed to address missing covariate data. The final data analysis was performed using R 3.4.3, EmpowerStats2.0, and EmpowerStats4.0. A two-sided p value less than 0.05 was deemed statistically significant.

This study involved secondary analysis of de-identified, publicly available data from the National Health and Nutrition Examination Survey (NHANES) 2011–2014. The original NHANES study protocol was reviewed and approved by the National Center for Health Statistics (NCHS) Research Ethics Review Board. Since our analysis used only pre-existing, anonymized data, no additional ethical approval from our institution was required. Written informed consent was obtained from all participants by NCHS during the original data collection. As the dataset contains no personally identifiable information, re-consent was not necessary for this analysis.

## Results

### Baseline characteristics

In this cross-sectional study of 5215 participants, the average age was 31.48 years with a standard deviation of 24.42. Males comprised 49.60% (n = 2587) of the sample, with a median blood cadmium concentration of 0.15 (IQR: 0.07–7.23). Table 1 presents the participants’ baseline characteristics categorized by blood cadmium levels. As shown in Table 1, individuals with the high blood cadmium group (cadmium level >0.24), demonstrated the following attributes: a large percentage of male, a higher proportion of older individuals, a great presence of non-Hispanic white individuals, and primarily current smokers. In comparison to the group with low blood cadmium group (cadmium level <0.1), the group with high cadmium levels exhibited higher BMI levels. Moreover, there was an increased occurrence of hypertension and diabetes.

**Table 1:** The demographic and clinical characteristics of the patients by tertiles of baseline lead.

Characteristics	Cadmium (ug/L)			P value
	Low (<0.10)	Middle (0.10–0.24)	High (>0.24)	
N	1733	1740	1742	
age (years)	10.3 ± 11.7	27.6 ± 21.2	50.2 ± 18.7	<0.001
Gender				<0.001
Male n (%)	933 (53.8%)	932 (53.6%)	722 (41.4%)	
Female n (%)	800 (46.2%)	808 (46.4%)	1020 (58.6%)	
Ethnicity				<0.001
Mexican American n (%)	370 (21.4%)	398 (22.9%)	201 (11.5%)	
Other Hispanic n (%)	206 (11.9%)	176 (10.1%)	130 (7.5%)	
Non-Hispanic White n (%)	561 (32.4%)	561 (32.2%)	726 (41.7%)	
Non-Hispanic Black n (%)	408 (23.5%)	363 (20.9%)	348 (20.0%)	
Other Race n (%)	188 (10.8%)	242 (13.9%)	337 (19.3%)	
PIR	1.8 ± 1.5	2.4 ± 1.7	2.4 ± 1.6	<0.001
BMI (kg/${m}^{2}$)	20.4 ± 6.8	25.3 ± 8.3	28.1 ± 7.0	<0.001
Vitamin D(nmol/L)	66.2 ± 18.9	62.8 ± 23.9	66.5 ± 28.3	<0.001
Current Smoking n (%)	29 (13.8%)	212 (22.0%)	943 (56.4%)	<0.001
Hypertension n (%)	56 (21.7%)	284 (27.2%)	675 (39.8%)	<0.001
Diabetes n (%)	21 (1.2%)	96 (5.5%)	208 (11.9%)	<0.001

BMI was calculated as the body weight in kilograms divided by the square of the height in meters.

*BMI* body mass index, *PIR* poverty income ratio.

### Associations between blood cadmium levels and periodontitis

Table 2 presents the association between blood cadmium levels and periodontitis risk. To study the independent effect of blood cadmium levels on periodontitis, we constructed three regression models. Following multivariate adjustments for factors such as age, gender, ethnicity, BMI, PIR, smoking status, vitamin D levels, diabetes, and hypertension (Model 3), the multivariate-adjusted OR with 95% confidence intervals (CI) for blood cadmium categories (<0.1, 0.10–0.24, >0.24 ug/L) were 1.00 (reference), 1.66 (0.84, 3.10), and 1.93 (1.00, 3.70), respectively.

**Table 2:** Relative odds of Periodontitis according to cadmium in different models.

Cadmium(ug/L)	Event (%)	Periodontitis, OR (95%CI)		
		Model1	Model2	Model3
Per 1unit increase	5215 (100%)	1.83 (1.52, 2.21)	2.15 (1.75, 2.64)	1.51 (1.22,1.86)
Tertiles				
Low (<0.10)	1733 (33.23%)	1	1	1
Middle (0.10–0.24)	1740 (33.37%)	1.30 (0.75, 2.25)	1.34 (0.76, 2.37)	1.38 (0.76,2.51)
High (>0.24)	1742 (33.40%)	2.06 (1.22, 3.49)	2.38 (1.37, 4.14)	1.71 (0.94,3.10)

Model 1 was adjusted for none.

Model 2 was adjusted for age, gender, and ethnicity.

Model 3 was adjusted for age, gender, ethnicity, PIR, BMI, smoking status, Vitamin D, Hypertension, Diabetes.

### The detection of nonlinear relationships

**Fig. 2:**
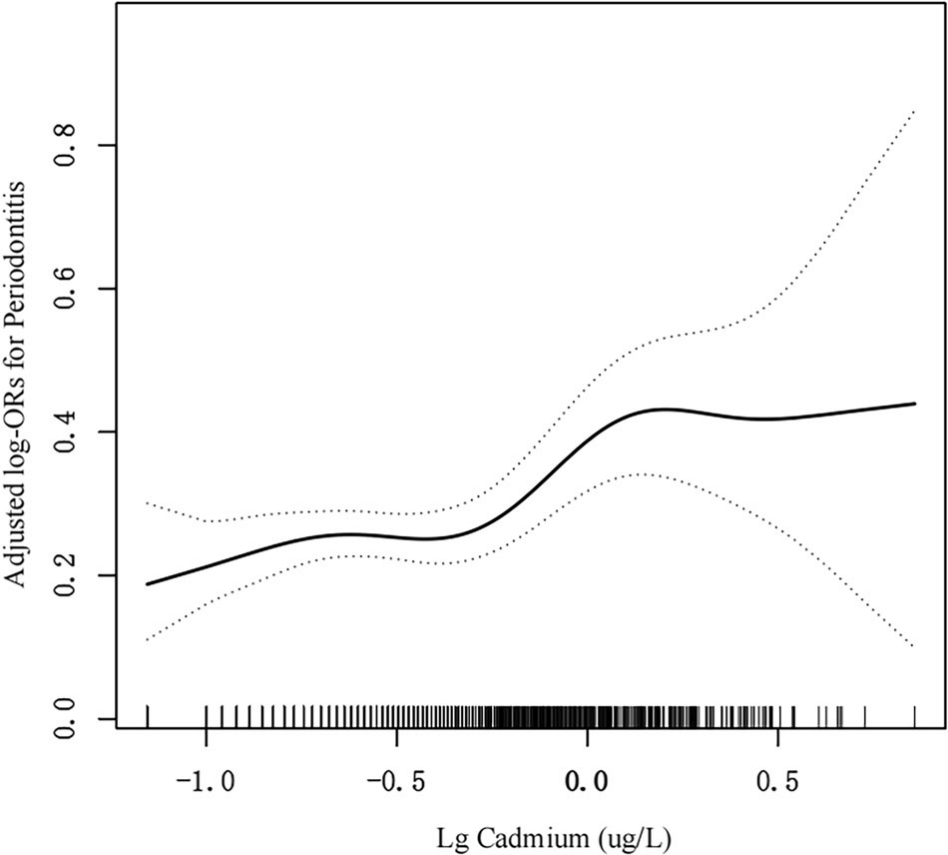
Association between blood cadmium levels with periodontitis. Adjusted for age, gender, ethnicity, PIR, BMI, smoking status, Vitamin D, Hypertension, Diabetes. The solid and dotted lines represent the estimated values and their corresponding 95% CIs, respectively.

Using generalized additive models and smoothed curve fitting, we identified nonlinear associations between blood cadmium concentrations and periodontitis (Fig. 2). We integrated a standard linear model with a two-piecewise linear model to explore the nonlinear association between blood cadmium levels and periodontitis. (P < 0.05 for log-likelihood ratio). The OR for periodontitis was 0.88 (95% CI: 0.31, 2.48; p = 0.81) for cadmium levels below 0.37 µg/L, 12.40 (95% CI: 2.77, 55.57; p < 0.001) for levels between 0.37 and 1.20 µg/L, and 0.45 (95% CI: 0.06, 3.39; p = 0.44) for levels above 1.20 µg/L (Table 3).

**Table 3:** Threshold effect analysis of blood cadmium levels in periodontitis patients.

Inflection points of cadmium(ug/L)	Adjusted OR (95% CI), P value
<0.37	0.88 (0.31, 2.48) 0.81
0.37–1.20	12.40 (2.77, 55.57) <0.001
>1.20	0.45 (0.06, 3.39) 0.44

Adjusted for age, gender, ethnicity, PIR, BMI, smoking status, Vitamin D, Hypertension, Diabetes.

In the supplementary analysis, the trend of the sensitivity analysis was consistent with that of the main analysis (Fig. S1). Multiple imputation techniques were employed to address missing covariate data. Similar results were obtained after considering the impact of missing data (Table S1).

## Discussion

This study found a nonlinear relationship between blood cadmium levels and periodontitis risk in U.S. adults. The key discovery was that the risk of periodontitis increased significantly when blood cadmium levels were between 0.37 and 1.20 µg/L. As far as we know, this is the first study to pinpoint the threshold at 0.37 and 1.20 µg/L. Earlier studies investigated the association between exposure to cadmium and periodontitis in different populations, utilizing data from the NHANES [19–21]. The first study by Arora et al. (2009) analyzed the relationship between environmental cadmium exposure and periodontal disease in 11,412 U.S. adults. The study found that higher urinary cadmium levels were associated with increased odds of periodontal disease (OR = 1.54), even after adjusting for tobacco exposure and other confounders [19]. The second study by Huang et al. (2022) focused on the association between blood levels of five trace minerals (selenium, manganese, lead, cadmium, and mercury) and periodontitis in 4964 U.S. adults. The results indicated that blood lead and cadmium levels were positively associated with CAL. The study also identified threshold and saturation effects for cadmium and lead, respectively, and highlighted stronger associations in males, the elderly, and individuals with diabetes [20]. The third study by Yang et al. (2024) examined the relationship between individual and combined exposures to 10 metals (including cadmium, lead, manganese, mercury, copper, zinc, calcium, potassium, sodium, and iron) and periodontitis in 2148 U.S. adults aged 30 years and older. The study found that elevated blood cadmium and lead levels were positively associated with periodontitis, with ORs of 1.73 and 1.17, respectively. The study also suggested that co-exposure to multiple metals was positively associated with periodontitis, potentially mediated by sex hormones and oxidative stress indicators [21].

Similarly, our studies consistently found that cadmium exposure, whether measured in blood or urine, was associated with an increased risk of periodontitis. Additionally, our study and Huang et al. (2022) [20] both identified nonlinear relationships, indicating that the effect of cadmium on periodontitis may depend on specific exposure ranges. The use of large, nationally representative samples further strengthens the generalizability of the findings across the U.S. adult population. These similarities underscore the robustness of the association between cadmium and periodontitis across different studies and methodologies. However, the key differences lie in the specific thresholds, risk magnitudes, and measurement methods. Our study identified distinct cadmium thresholds (0.37 µg/L and 1.20 µg/L) with a significantly higher odds ratio (OR = 12.40) within the 0.37–1.20 µg/L range, whereas other studies reported lower ORs (e.g., 1.73 in Yang et al. and 1.54 in Arora et al.) [19, 21] without such precise thresholds. This discrepancy may stem from differences in statistical approaches, as our study employed nonlinear models to identify specific exposure windows, while others used linear or logistic regression. Additionally, our study measured blood cadmium, reflecting recent exposure, whereas Arora et al. used urinary cadmium, which indicates long-term exposure [19]. Population characteristics, such as age distribution and underlying health conditions, as well as variations in confounding factors controlled for in the analyses, could also contribute to these differences. These variations highlight the complexity of the cadmium-periodontitis relationship and the need for further research to clarify the dose-response dynamics and underlying mechanisms.

Cadmium is a widely recognized environmental pollutant known for its various detrimental effects on human health, particularly in relation to periodontal disease. Recent studies have indicated a correlation between cadmium exposure and the onset and progression of periodontal disease, suggesting that cadmium may influence oral health through multiple mechanisms [23, 24]. Firstly, cadmium exposure can induce oxidative stress and inflammatory responses, which are critical factors in the destruction of periodontal tissues. The generation of reactive oxygen species (ROS) as a result of cadmium exposure has been well documented, contributing to tissue damage and inflammation in periodontal disease [25, 26]. Elevated levels of inflammatory mediators, such as interleukin-6 (IL-6) and tumor necrosis factor-alpha (TNF-α), have been observed in individuals exposed to cadmium, further supporting its role in promoting periodontal inflammation [11]. Secondly, the toxic effects of cadmium may impair the function of periodontal ligament fibroblasts and osteoblasts, which are essential for the regeneration and repair of periodontal tissues. Cadmium has been shown to disrupt cellular functions, leading to decreased viability and altered activity of these critical cell types [26]. This impairment can hinder the healing processes necessary for maintaining periodontal health, thereby exacerbating the progression of periodontal disease. Furthermore, cadmium exposure may interfere with the normal functioning of the immune system, weakening the body’s defense against oral bacterial infections. This immunosuppressive effect can increase susceptibility to periodontal pathogens, contributing to the development and exacerbation of periodontal disease [11]. Moreover, the interaction between cadmium and other environmental factors, such as smoking, has been shown to further compromise oral health. Studies have indicated that smoking, which is a known risk factor for periodontal disease, can exacerbate the toxic effects of cadmium, leading to a more severe inflammatory response and increased periodontal tissue destruction [10, 11]. The cumulative effects of these interactions underscore the importance of considering heavy metal exposure, including cadmium, as a significant risk factor in the pathogenesis of periodontal disease.

### Study limitations

Our research encountered several limitations. First, one common issue in observational research is the existence of unmeasured confounders. Furthermore, although our multivariate models adjusted for smoking status, residual confounding may remain. Stratified analyses by smoking history (never, former, current) could help further clarify the association. Unfortunately, such analyses were not feasible in the present study due to sample size constraints within subgroups. Thus, it is important to carry out large-scale prospective studies in the future to validate the results shown in this study. Second, our study contains incomplete data for certain variables. Nevertheless, we used contemporary methods to deal with missing data to minimize bias. Third, it is noteworthy that the potential resulting from interventions would bias towards to the null and thus result in an underestimation of the association between blood cadmium levels and periodontitis. Finally, we acknowledge that the participants were patients referred to the U.S adults for some reason, limiting the generalization of the findings to other populations.

## Conclusions

This study identified a nonlinear association between blood cadmium levels and periodontitis risk in U.S. adults. The risk of periodontitis increased significantly when blood cadmium levels were between 0.37 and 1.20 µg/L. However, due to the cross-sectional design, the presence of potential unmeasured confounders, missing data, and limited generalizability, these findings should be interpreted with caution. Future large-scale prospective studies in diverse populations are needed to confirm the causal relationship and clarify the underlying mechanisms linking cadmium exposure to periodontal disease.

## Supplementary information

Supplementary Figure S1

Supplementary Table S2

## Data Availability

This study analyzed publicly available datasets. The data is available at http:// www.cdc.gov/nchs/nhanes/.
