# Supplementary material for: Nonlinear association between blood cadmium levels and periodontitis: a cross-sectional study from NHANES 2011–2014
Source: BDJ Open. 2025 Nov 13;11:85. doi: 10.1038/s41405-025-00376-y (PMC12615595; doi:10.1038/s41405-025-00376-y)
Supplement: Supplementary file 1 — Supplementary Figure S1 [file 41405_2025_376_MOESM1_ESM.pdf]

Figure S1 Forest plots of stratified analysis of cadmium with periodontitis.

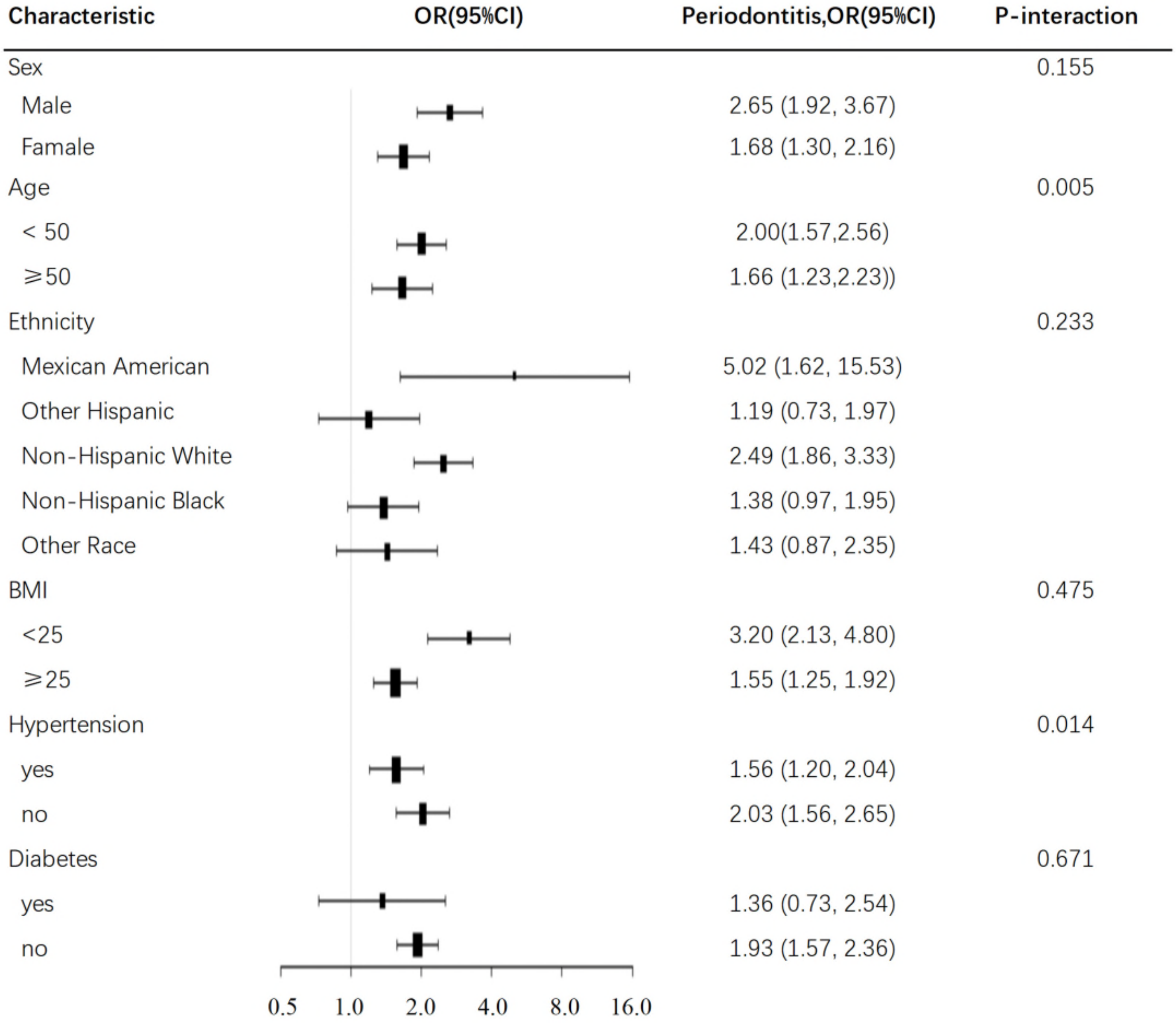

Characteristic	Periodontitis,OR(95%CI)	P-interaction
Sex		0.155
Male	2.65 (1.92, 3.67)	
Famale	1.68 (1.30, 2.16)	
Age		0.005
< 50	2.00(1.57,2.56)	
⩾50	1.66 (1.23,2.23))	
Ethnicity		0.233
Mexican American	5.02 (1.62, 15.53)	
Other Hispanic	1.19 (0.73, 1.97)	
Non-Hispanic White	2.49 (1.86, 3.33)	
Non-Hispanic Black	1.38 (0.97, 1.95)	
Other Race	1.43 (0.87, 2.35)	
BMI		0.475
<25	3.20 (2.13, 4.80)	
⩾25	1.55 (1.25, 1.92)	
Hypertension		0.014
yes	1.56 (1.20, 2.04)	
no	2.03 (1.56, 2.65)	
Diabetes		0.671
yes	1.36 (0.73, 2.54)	
no	1.93 (1.57, 2.36)	

Note: Age, gender, race/ethnicity, PIR, BMI, smoking status, Vitamin D, Hypertension, Diabetes were all adjusted except the variable itself.

Abbreviations: OR: odd ratio; CI: confidence interval
