# Supplementary material for: Nonlinear association between blood cadmium levels and periodontitis: a cross-sectional study from NHANES 2011–2014
Source: BDJ Open. 2025 Nov 13;11:85. doi: 10.1038/s41405-025-00376-y (PMC12615595; doi:10.1038/s41405-025-00376-y)
Supplement: Supplementary file 2 — Supplementary Table S2 [file 41405_2025_376_MOESM2_ESM.docx]

**Table S2** Logistic regression analyses of the associations between cadmium and periodontitis

|  | Non-adjusted | Adjust I (Complete case) | Adjust II (Multiple imputation) |
| --- | --- | --- | --- |
|  | 1.83 (1.52, 2.21) <0.05 | 1.51 (1.22, 1.86) <0.05 | 1.51 (1.21, 1.86) <0.05 |
| Cadmium quartile |  |  |  |
| Low | 1.0 | 1.0 | 1.0 |
| Middle | 1.30 (0.75, 2.25) 0.35 | 1.38 (0.76, 2.51) 0.29 | 1.39 (0.76, 2.52) 0.28 |
| High | 2.06 (1.22, 3.49) <0.05 | 1.71 (0.94, 3.10) 0.08 | 1.71 (0.94, 3.11) 0.08 |

Note: Adjusted for age, gender, race/ethnicity, PIR, BMI, smoking status, Vitamin D, Hypertension, Diabetes

Abbreviations: OR: odd ratio; CI: confidence interval
